# Predicting visual acuity in Bietti crystalline dystrophy: evaluation of image parameters

**DOI:** 10.1186/s12886-021-01811-y

**Published:** 2021-02-04

**Authors:** Chu-Yen Huang, Eugene Yu-Chuan Kang, Lung-Kun Yeh, An-Lun Wu, Pei-Kang Liu, I-Wen Huang, Joseph Ryu, Laura Liu, Wei-Chi Wu, Chi-Chun Lai, Kuan-Jen Chen, Nan-Kai Wang

**Affiliations:** 1 Department of Ophthalmology, Chang Gung Memorial Hospital, Linkou Medical Center, Taoyuan, Taiwan; 2 College of Medicine, Chang Gung University, Taoyuan, Taiwan; 3 Department of Ophthalmology, Mackay Memorial Hospital, Hsinchu, Taiwan; 4 Department of Ophthalmology, Edward S. Harkness Eye Institute, Columbia University Irving Medical Center, Columbia University, New York, USA; 5 Department of Ophthalmology, Kaohsiung Medical University Hospital, Kaohsiung, Taiwan; 6 Department of Ophthalmology, School of Medicine, Kaohsiung Medical University, Kaohsiung, Taiwan; 7 Institute of Biochemical Science, National Sun Yat-Sen University, Kaohsiung, Taiwan; 8 Department of Ophthalmology, Chang Gung Memorial Hospital, Keelung, Taiwan

**Keywords:** Best corrected visual acuity (BCVA), Bietti crystalline dystrophy (BCD), Choroidal thickness, Crystalline, Ellipsoid zone disruption, Fundus autofluorescence (FAF), Near-infrared reflectance (NIR), Sclerotic vessels, Spectral domain-optical coherence tomography (SD-OCT)

## Abstract

**Background:**

To analyze multiple imaging modalities in patients with Bietti crystalline dystrophy (BCD) and to investigate which factors from these modalities are associated with best corrected visual acuity (BCVA).

**Methods:**

In this retrospective study, 40 eyes from 22 patients with BCD were included and were separated into group 1 (BCVA ≤20/200) and group 2 (BCVA > 20/200). Data including BCVA and characteristic findings from near-infrared reflectance (NIR) imaging, fundus autofluorescence (FAF), and spectral domain-optic coherence tomography (SD-OCT) were analyzed and compared. The outcome measures of multimodal imaging were evaluated for correlation with BCVA.

**Results:**

NIR is a good diagnostic tool for detecting either crystalline or sclerotic vessels in BCD. Patients in group 1 tended to have a thinner choroid (*P* = 0.047) with ellipsoid zone (EZ) disruption (*P* = 0.011). Calculation of the area under the curve indicated that EZ disruption detected on SD-OCT could be a good predictor of legal blindness in BCD.

**Conclusion:**

For the diagnosis of BCD, NIR could be a good diagnostic tool. Of the studied imaging modalities, we found that EZ disruption at the fovea were strongly associated with legal blindness, which could be easily assessed by SD-OCT.

**Supplementary Information:**

The online version contains supplementary material available at 10.1186/s12886-021-01811-y.

## Background

Bietti crystalline dystrophy (BCD) (OMIM#210370) is a severe inherited retinal dystrophy that is caused by autosomal recessive mutations in the *cytochrome P450 family 4 subfamily V member 2* (*CYP4V2*) gene [1]. The CYP4V2 enzyme is involved in fatty acid oxidation, and the protein is highly expressed in retinal pigment epithelium (RPE) cells. As such, it is postulated that CYP4V2 defects affect lipid breakdown [2]; however, the precise pathology of how these mutations lead to photoreceptor degeneration remains unknown. Given our limited understanding of disease pathogenesis, there is currently no available treatment for BCD.

The disease was first described by Bietti in 1937 based on three patients who exhibited numerous crystal deposits in the retina and perilimbal cornea. Additional clinical characteristics include nyctalopia and visual field defects that manifest in the second to fourth decade of life [3]. As the disease progresses, crystal deposits gradually decrease in number [4], and retinal and choroid atrophy develop together with progressive visual loss [5]. To date, several studies have characterized the clinical features of BCD using fundus photography [6], fluorescence angiography, fundus autofluorescence (FAF), spectral domain-optical coherence tomography (SD-OCT) and near-infrared reflectance (NIR) imaging [7]. Although fundus photography, FAF and SD-OCT have been used to classify different stages of BCD [8], no study has analyzed the correlation of these image characteristics with visual function. Recently, Miyata et al. reported a correlation between BCD features on OCT/optical coherence tomography angiography (OCTA) and visual function including visual acuity (VA) and visual field (VF) [9]. However, it is unknown whether features observed in other multimodal imaging techniques such as color fundus, FAF and NIR could be used as predictors of visual function.

The purpose of this study is to compare the results of several retinal imaging modalities in BCD patients and identify indicators of visual function. Such indicators from noninvasive imaging modalities can provide a thorough assessment of retinal structure and progression of the disease. Several clinical trials of gene therapy for inherited retinal dystrophies have been initiated after the United State Food and Drug Administration approved the first RPE65 gene therapy [10]. Although there is currently no definite treatment for BCD, reliable indicators that correlate with visual function and capture changing structural and functional features of BCD may serve as potential tools for monitoring disease progression as well as valuable outcome measures for future gene therapy in interventional trials.

## Methods

### Study population

We retrospectively reviewed patients with BCD who visited the Department of Ophthalmology at Chang Gung Memorial Hospital in Linkou Medical Center between January 2010 and December 2018. The study was approved by the Institutional Review Board of Chang Gung Memorial Hospital, Taiwan (IRB 202001456B0). BCD was clinically diagnosed based on patients’ symptoms and retinal images. The characteristic features of BCD include the presence of crystalline deposits at the posterior pole, prominent sclerotic choroidal vessels, choroid–retinal dystrophy, patchy atrophy of FAF, with or without outer retinal tubulation (ORT) on SD-OCT.

### Clinical examination

All the patients underwent a complete ocular examination at their first visit, including measurement of BCVA, refractive error, intraocular pressure, dilated fundus examination, fundus photography, SD-OCT, NIR imaging and FAF imaging. Some patients also received fluorescence angiography, a static VF test, blue-light full-field electroretinogram, or genetic testing for *CYP4V2* mutations. NIR and FAF were performed with a confocal scanning laser ophthalmoscope (Heidelberg Retina Angiograph HRA2; Heidelberg Engineering, Dossenheim, Germany). SD-OCT images were obtained using a Spectralis HRA-OCT system (Heidelberg Engineering). Patients with other ocular diseases that could also attribute to prominently decreased vision were excluded.

### Acquisition of Image Biometrics

The results of the imaging modalities analyzed in our study were collected on the first visit for each patient. All of the characteristics of each imaging modality were classified by at least three independent investigators blinded to the associated BCVA. The presence of crystalline deposits and sclerotic vessels at the posterior pole was determined on either fundus photography or NIR. Foveal retinal thickness (average thickness at 2 mm diameter from the center of the fovea), subfoveal choroidal thickness, ellipsoid zone (EZ) line integrity at the fovea, and the presence of outer retinal tubulation (ORT) were identified from the SD-OCT images. The presence or absence of ORT was determined at the center of a horizontal and vertical 8.0 mm SD-OCT scan cross. The severity of changes in FAF images was graded as described by Li et al. in 2015 [8], based on the staging system established by Yuzawa et al. [6] Stage 1 was defined as normal AF or hyper/hypo-AF spots confined to the posterior pole. Stage 2 was defined as confluent hypo-AF patches extending beyond the posterior pole. Stage 3 was defined as the clear absence of AF at the posterior pole. Retinal and choroidal biometry was measured using the built-in software in the SD-OCT system (Heidelberg Engineering). Subfoveal choroidal thickness was defined as the distance between the outer borders of the RPE and the hyperrefractive line behind the large vessel layers of the choroid and was measured manually at the subfovea using build-in software by two independent investigators.

### Statistical analysis

All the eyes were divided into two groups according to BCVA at first visit. Group 1 consists of patients with BCVA ≤20/200, and group 2 includes patients with BCVA > 20/200. BCVA ≤20/200 was defined as legal blindness according to National Eye Institute of the US [11].

Statistical analysis was performed using SPSS (IBM SPSS Statistics for Windows, Version 21.0. IBM Corp. Armonk, NY). Continuous covariates were assessed using an independent *t* test. A Mann–Whitney *U* test was performed for those covariates that were not normally distributed. Categorical covariates were assessed using the Pearson chi-squared test, or with Fisher’s exact test if the expected value was < 5. Univariate regression was calculated for variations in BCVA relative to age, sex, crystalline deposits, central retinal thickness, subfoveal choroidal thickness, EZ disruption at the fovea, ORT, sclerotic vessels, and FAF staging. Multiple logistic regression was applied to evaluate the explanatory variables with regard to the dependent variable, including age, subfoveal choroidal thickness and EZ disruption at the fovea. The cutoff values of these biometrics that predicted BCVA ≤20/200 were analyzed using a receiver-operating characteristic (ROC) curve. The accuracy was measured by the area under the curve (AUC). An AUC of 1.0 represents a perfect test for discrimination, whereas an AUC of 0.5 indicates random assignment. Statistical significance was defined as a *P-*value < 0.05.

## Results

### Patient demographics

**Fig. 1:**
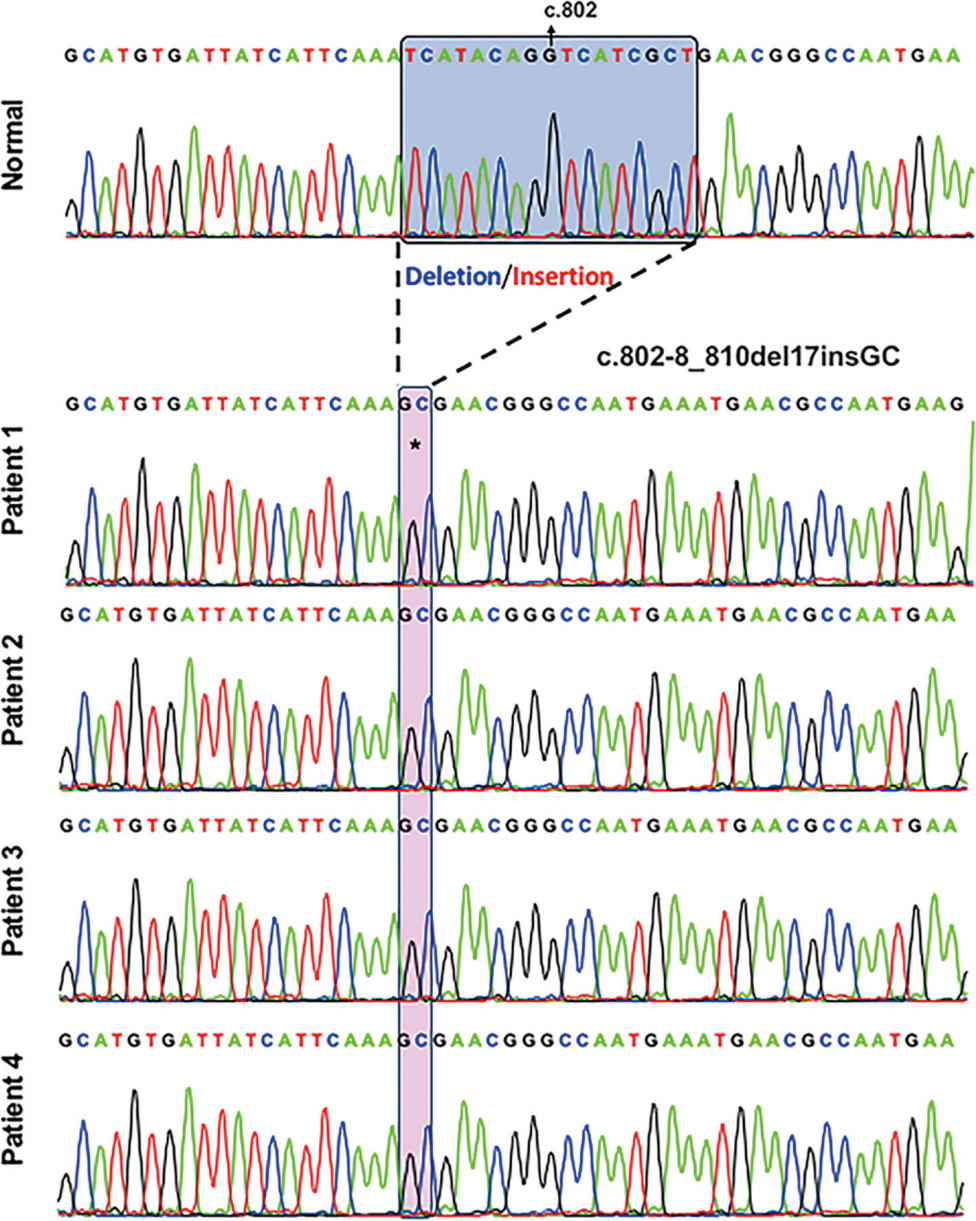
Genetic analysis. 4 patients in our study received genetic analysis, and all of them had homozygous mutation c.802–8_810del17insGC in *CYP4V2* gene. The blue box indicates the deletion of 17 nucleotides from intron position 802–8 to exon position 810 (coding sequence), and the red box showed that GC was inserted instead

In total, 40 eyes from 22 patients with BCD were included and analyzed in our study. Patients with choroidal neovascularization (2 eyes), glaucoma (1 eye), and macula scar (1 eye) were excluded. Among these 22 patients clinically diagnosed with BCD, four had previously undergone genetic testing from a prospective study (Genetic study in hereditary retinal and optic nerve diseases, IRB 201601569B0C601), all of whom were confirmed to have the homozygous mutation c.802–8_810del17insGC in *CYP4V2* gene (Fig. 1), which is the most common pathogenic variant of BCD in Eastern countries [12, 13]. Of the 40 eyes, 14 had a BCVA ≤20/200 and were classified as group 1 (35%), while 26 had a BCVA > 20/200 and were classified as group 2 (65%). The mean age of the participants was 46.80 ± 12.2 years (mean ± SD). The mean follow-up period was 30.7 ± 28.39 months (mean ± SD).

### Features in imaging modalities

**Fig. 2:**
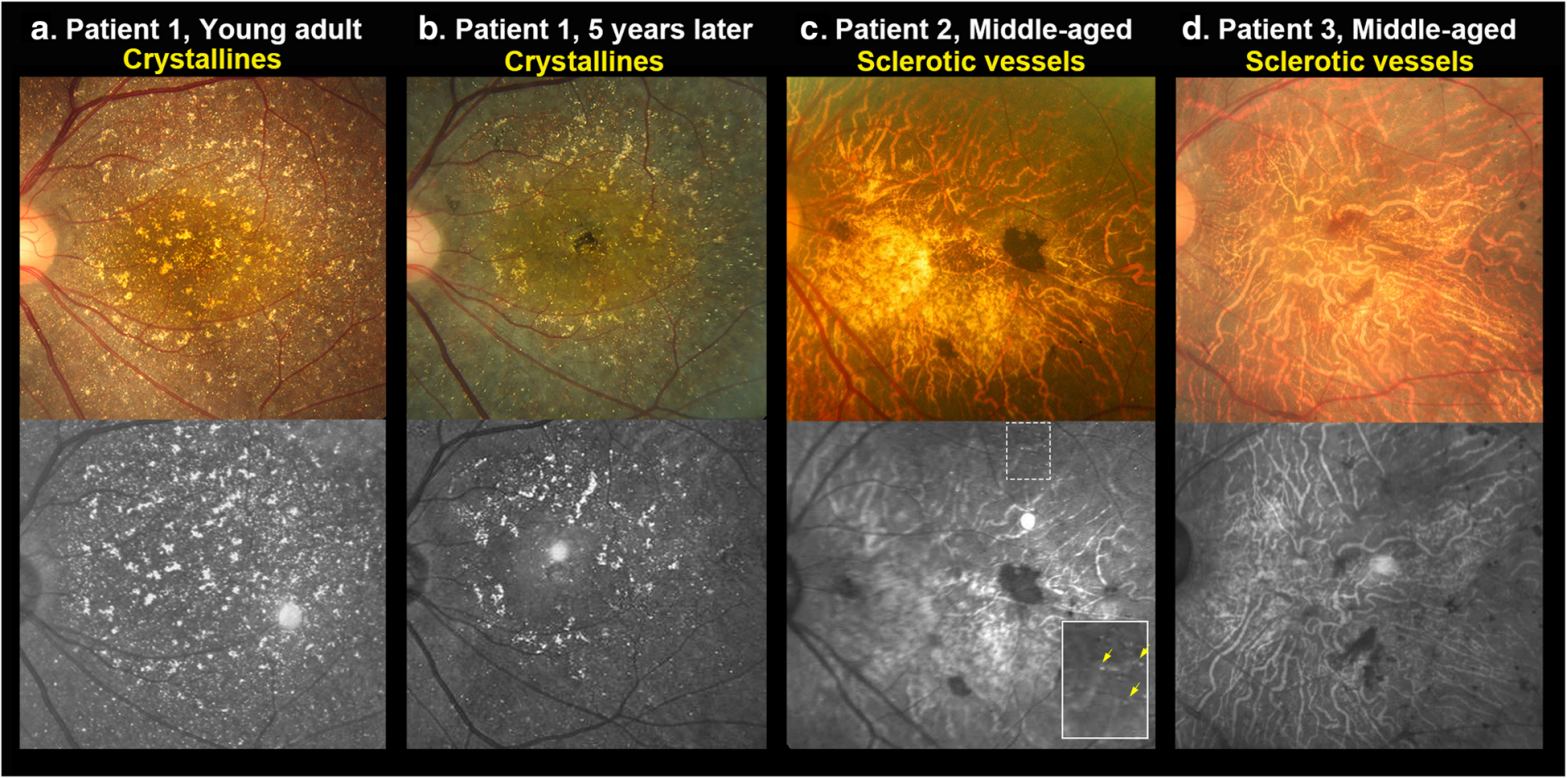
NIR imaging. **a** Prominent hyperreflective crystalline deposits without sclerotic choroid vessels. **b** The color fundus and NIR images of the same patient after 5 years showing decreased crystalline deposits. There are still no sclerotic vessels on her NIR image. **c** Prominent sclerotic vessels have appeared. There are much fewer crystalline deposits. Some deposits are shown in the white dotted square (yellow arrows). **d** NIR image of advanced-stage BCD showing prominent sclerotic choroidal vessels and no crystalline deposits

**Fig. 3:**
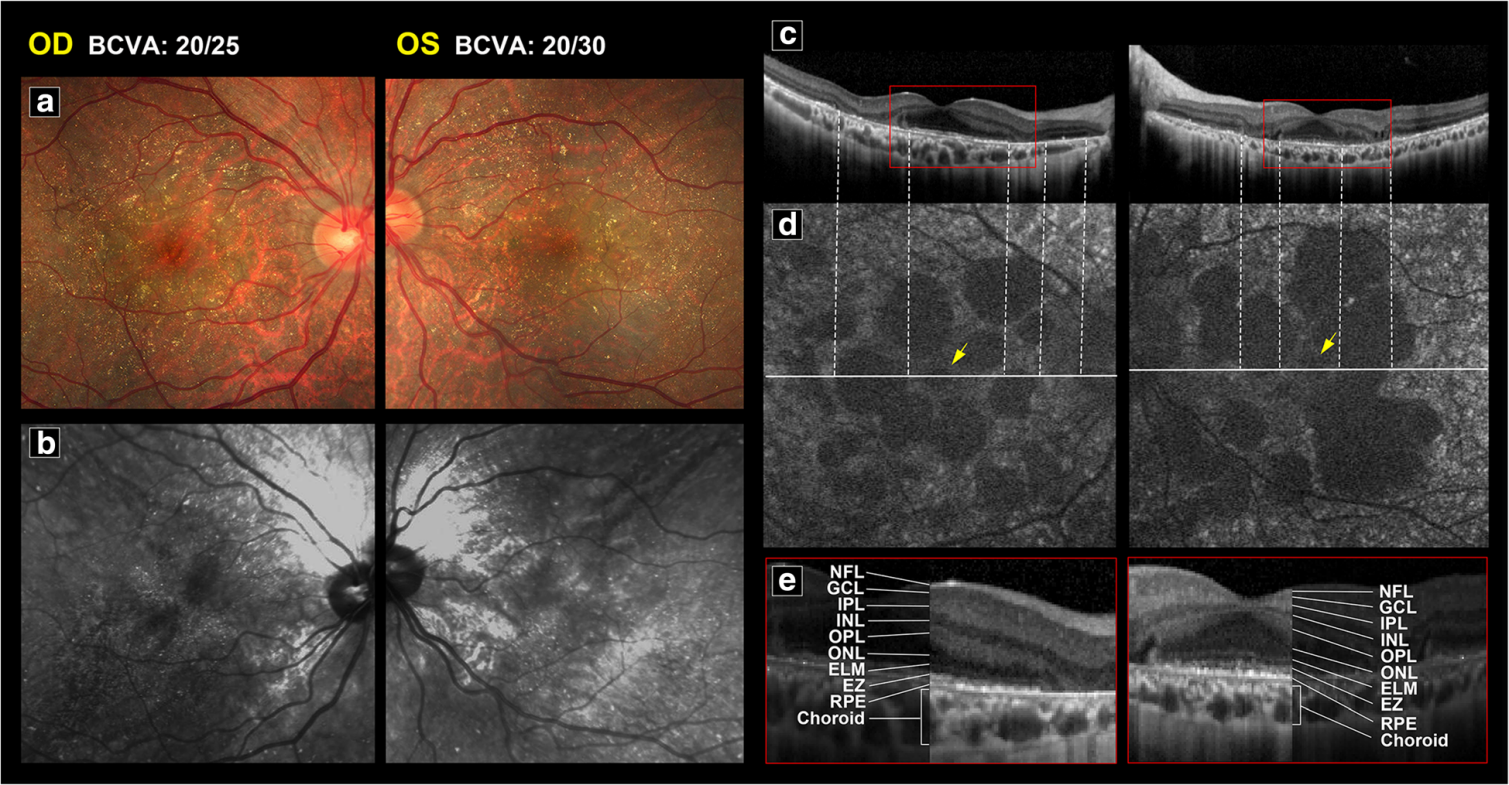
An young adult with a diagnosis of BCD, BCVA of 20/22 (right eye, Oculus Dexter (OD)) and 20/29 (left eye, Oculus Sinister (OS)). **a** Color fundus showing obvious crystalline deposits. **b** Crystalline deposits can be clearly seen on NIR imaging. **c** OCT revealed preserved retinal structure near the fovea (red square) in both eyes. Outside the preserved island, outer retinal layer (ONL) destruction and EZ loss were observed. **d** In this case, some hypo-AF patches on FAF imaging corresponded to ONL and EZ destruction seen on OCT. Relatively low AF at the fovea (yellow arrows) corresponded to normal structures seen on OCT. **e** Enlarging the central island on SD-OCT, there are intact retinal structures in the central area (l *NFL* nerve fiber layer; *GCL *ganglion cell layer; *IPL* inner plexiform layer; *INL* inner nulear layer; *OPL* outer plexiform layer; *ONL* outer nuclear layer; *ELM* external limiting membrane; *EZ* ellipsoid zone; *RPE* retinal pigment epithelium)

**Fig. 4:**
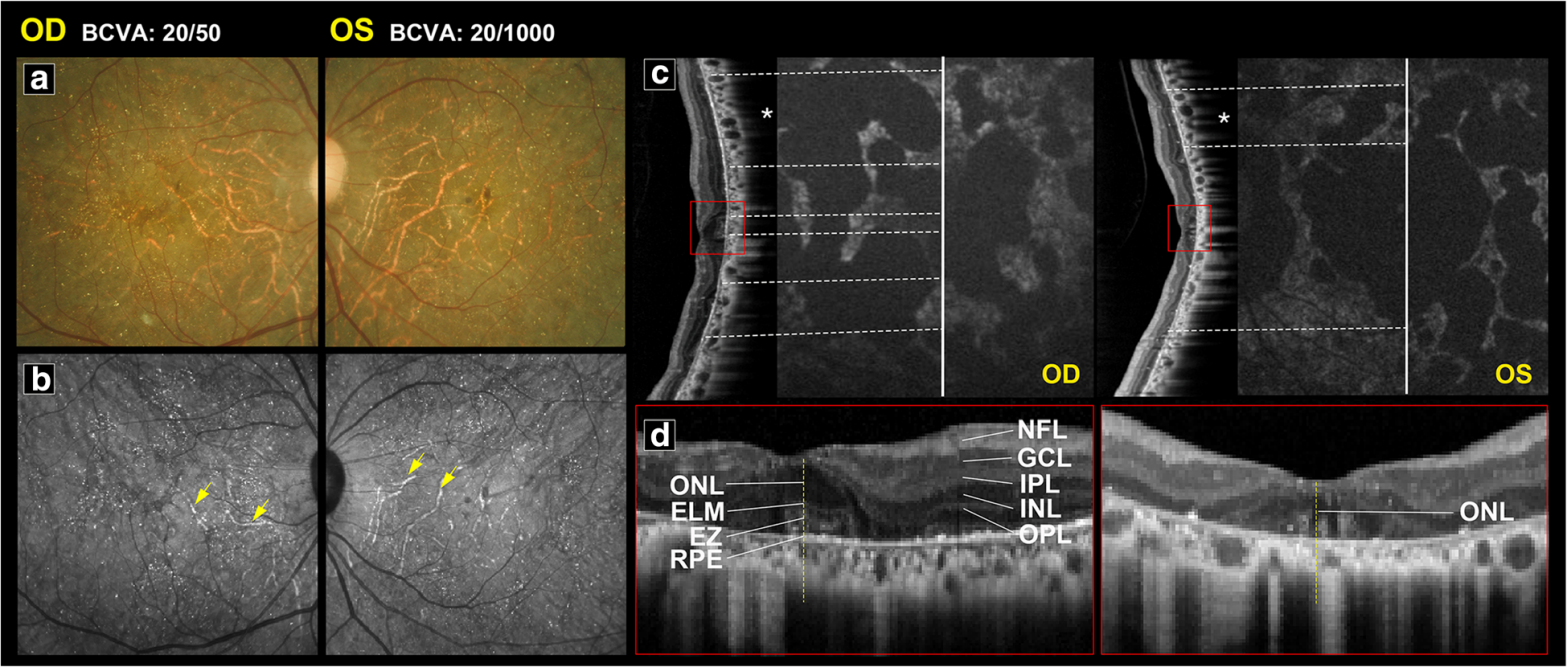
This middle-aged patient had BCVA of 20/50 (OD) and 20/1000 (OS). **a** Color fundus revealed a greenish background color with depigmentation. **b** Obvious sclerotic vessels were evident on NIR (yellow arrows). Multiple hyperreflective crystal deposits could still be observed. **c** On OCT, a small foveal preservation island was demonstrated in the right eye. OCT of the left eye showed destruction of EZ and ONL. A normal/hyper-AF area on AF cannot predict structural preservation on OCT. The white star indicates the area of outer nuclear layer thinning and EZ disruption seen on OCT, but it corresponded to neither hypo-AF nor normal AF. **d** The foveal structures are labeled in high magnification from red squares in (**c**) (*NFL* nerve fiber layer; *GCL* ganglion cell layer; *IPL* inner plexiform layer; *INL* inner nulear layer; *OPL* outer plexiform layer; *ONL* outer nuclear layer; *ELM* external limiting membrane; *EZ* ellipsoid zone; *RPE* retinal pigment epithelium)

NIR can easily identify crystalline deposits and sclerotic vessels (Figs. 2, 3a, b, 4a, b). The crystalline deposits decreased in number as the disease progressed (Fig. 2a, b) and nearly disappeared in advanced stages of disease (Fig. 2d). By contrast, sclerotic choroidal vessels were absent during the early stages of disease (Fig. 2a, b) but became more prominent later (Fig. 2c, d). In our patients, all five eyes without crystalline deposits on their fundus had prominent sclerotic choroid vessels on their NIR images, which indicated later stages of BCD.

Using SD-OCT, disruption of the EZ can be observed in the affected area (Fig. 3c). These lesions initially presented as random patches with disruption of the EZ, followed by abrupt outer nuclear layer destruction. The retinal layers near the fovea (Fig. 3c, e) were usually preserved on SD-OCT until later stages of disease. Figure 4 shows examples of a patient with bilateral symmetric amounts of crystalline deposit and sclerotic vessels on color fundus (Fig. 4a) and NIR (Fig. 4b), similar FAF stages (Fig. 4c), and similar subfoveal choroidal thickness in both eyes. However, one eye had a preserved EZ at the fovea while the other eye did not. The BCVA of the eye with a disrupted EZ at the fovea was 20/1000, which is considered to be legally blind, whereas BCVA of the other eye with a preserved island at the fovea was 20/50.

**Fig. 5:**
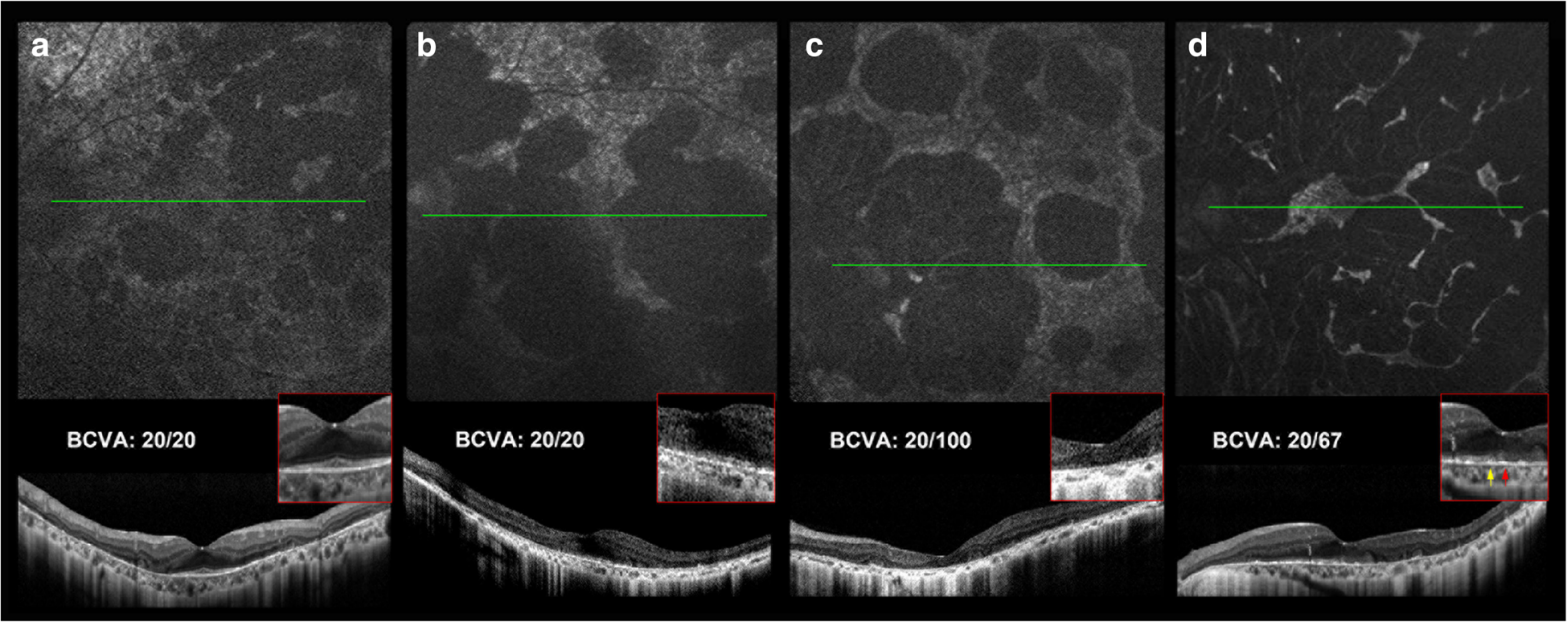
It is hard to recognize a central island on FAF because of the hypo-AF at the fovea. **a**, **b** The patients with preserved foveal structures, especially EZ, on OCT had a BCVA of 20/20. **c** OCT shows central destruction of the EZ. This eye had a BCVA of 20/100. On FAF, it was relatively difficult to determine whether the central area was preserved. **d** The preserved island of the normal retinal structure was eccentric. On OCT, a red arrow indicates the central fovea, but the EZ starts from the site where the yellowish arrow indicates the nasal retina. FAF shows confluent patches. The BCVA of this eye was 20/67 (*FAF* fundus autofluorescence; *OCT* optical choherence tomography; *BCVA* best corrected visual acuity; *EZ *ellipsoid zone)

FAF in BCD initially presented with some hyper- and hypo-AF spots. These hypo-AF spots gradually enlarged in size to hypo-AF patches and eventually became confluent (Fig. 5). A hypo-AF signal indicates RPE atrophy or RPE death and usually appears together with a disrupted EZ on SD-OCT, indicating photoreceptor degeneration. However, in some cases, we found that the area of disrupted EZ on SD-OCT did not necessarily correspond to the hypo-AF patches (Fig. 4c, white stars).

### Comparison of the two groups

The comparison of the two study groups is summarized in Table 1. The mean age of group 1 was 52.21 ± 13.47 years and that of group 2 was 43.88 ± 10.60 years (*P* = 0.038, independent t test). Refractive errors were − 2.26 D ± 2.65 D in group 1, and − 3.72 D ± 3.78 D in group 2 (*P* = 0.589, Mann-Whitney U test). 25 eyes in group 2 (96%) had crystalline deposits on the fundus, but crystals could only be observed in 10 eyes in group 1 (71%) (*P* = 0.043, Fisher’s exact test). The mean central retinal thickness was 256.86 μm ± 39.40 μm in group 1 and 272.73 μm ± 57.86 μm in group 2 (*P* = 0.366, independent t test). The mean subfoveal choroidal thickness was 73.93 μm ± 43.31 μm in group 1 and 126.20 μm ± 50.02 μm in group 2 (*P* = 0.002, independent t test). 13 eyes in group 1 (92.9%) and 5 eyes in group 2 (19.2%) showed EZ disruption at the fovea (*P* < 0.001, Fisher’s exact test).

**Table 1:** Summary of clinical and demographic characteristics of patients in Group 1 and Group 2

Variables	All patients		Group 1 (BCVA ≤ 20/200) (***N*** = 14)		Group 2 (BCVA > 20/200) (***N*** = 26)		***p***-value
	Mean	SD	Mean	SD	Mean	SD	
Age	46.80	12.20	52.21	13.47	43.88	10.60	*0.038
Follow-up (months)	30.70	28.39	28.27	27.99	35.21	29.61	0.747
Refractive error(D)	−3.29	3.51	−2.26	2.65	−3.72	3.78	0.589
Central retinal thickness (μm)	267.18	52.18	256.86	39.40	272.73	57.86	0.366
Subfoveal Choroidal thickness (μm)	107.91	53.54	73.93	43.31	126.20	50.02	*0.002
	N	%	N	%	N	%	
Male	15	37.50	4	28.60	11	42.30	0.502
Crystalline deposits	35	87.50	10	71.40	25	96.20	*0.043
EZ disruption at fovea	18	45.00	13	92.90	5	19.20	* < 0.001
Outer retinal tubulation	20	50.00	5	35.70	15	57.70	0.185
Sclerotic vessels	35	87.50	13	92.90	22	84.60	0.640
FAF staging							0.122
Stage 1	5	12.50	1	7.10	4	15.40	0.64
Stage 2	17	42.50	5	35.70	12	46.20	0.524
Stage 3	11	27.50	7	50.00	4	15.40	*0.029

*BCVA* best corrected visual acuity, *SD* standard deviation, *N* number, *EZ* ellipsoid zone, *FAF* fundus autofluorescence

*p < 0.05

Regarding the presence of ORT, 5 eyes in group 1 (35%) and 15 eyes in group 2 (57%) had ORT that was detected in either horizontal and/or vertical 8.0-mm scanning at the center SD-OCT (*P* = 0.185). Sclerotic vessels were observed on NIR images in 13 eyes in group 1 (93%) and 22 eyes in group 2 (85%) (*P* = 0.137, Pearson Chi-square). FAF images were classified into three stages. In group 1, only 1 eye (7%) was classified as stage 1, 5 eyes were classified as stage 2 (36%), and 7 eyes were classified as stage 3 (50%). In group 2, 4 eyes were classified as stage 1 (15%), 12 eyes were classified as stage 2 (46%), and 4 eyes were classified as stage 3 (15%) (*P* = 0.122, Pearson Chi-square).

### Statistics

The univariate logistic regression analysis for legal blindness is shown in Table 2. Parameters including sex, refractive errors, central retinal thickness, presence of ORT, sclerotic vessels and FAF staging were not correlated significantly with legal blindness. On the other hand, older age, decreased subfoveal choroidal thickness, EZ disruption at the fovea, and FAF stage 3 were correlated significantly with legal blindness.

**Table 2:** Univariate and multivariate analysis of BCVA for Group 1 and Group 2

	Univariate analysis				Multivariate analysis			
Variables	OR	95% CI		*p*-value	OR	95% CI		*p*-value
Age	1.064	1.001	1.131	*0.047	0.751	0.551	1.023	0.069
Follow-up (months)	1.009	0.986	1.032	0.457				
Refractive error(D)	1.149	0.896	1.473	0.275				
Central retinal thickness (μm)	0.994	0.98	1.007	0.359				
Subfoveal Choroidal thickness (μm)	0.975	0.957	0.993	*0.007	0.904	0.819	0.999	*0.047
Male	0.545	0.135	2.204	0.395				
Crystalline deposits	10	0.992	100.921	0.051				
EZ disruption at fovea	0.018	0.002	0.175	* < 0.001	0.002	0	0.234	*0.011
Outer retinal tubulation	2.455	0.642	9.391	0.19				
Sclerotic vessels	0.423	0.043	4.203	0.463				
FAF staging								
Stage 1	0.423	0.043	4.203	0.463				
Stage 2	1.543	0.405	5.879	0.525				
Stage 3	5.5	1.234	24.506	*0.025	0.076	0.003	2.069	0.126

*BCVA* best corrected visual acuity, *OR* odds ratio, *CI* confidence interval, *Sig*. significance; *EZ* ellipsoid zone, *FAF* fundus autofluorescence

*p < 0.05

**Fig. 6:**
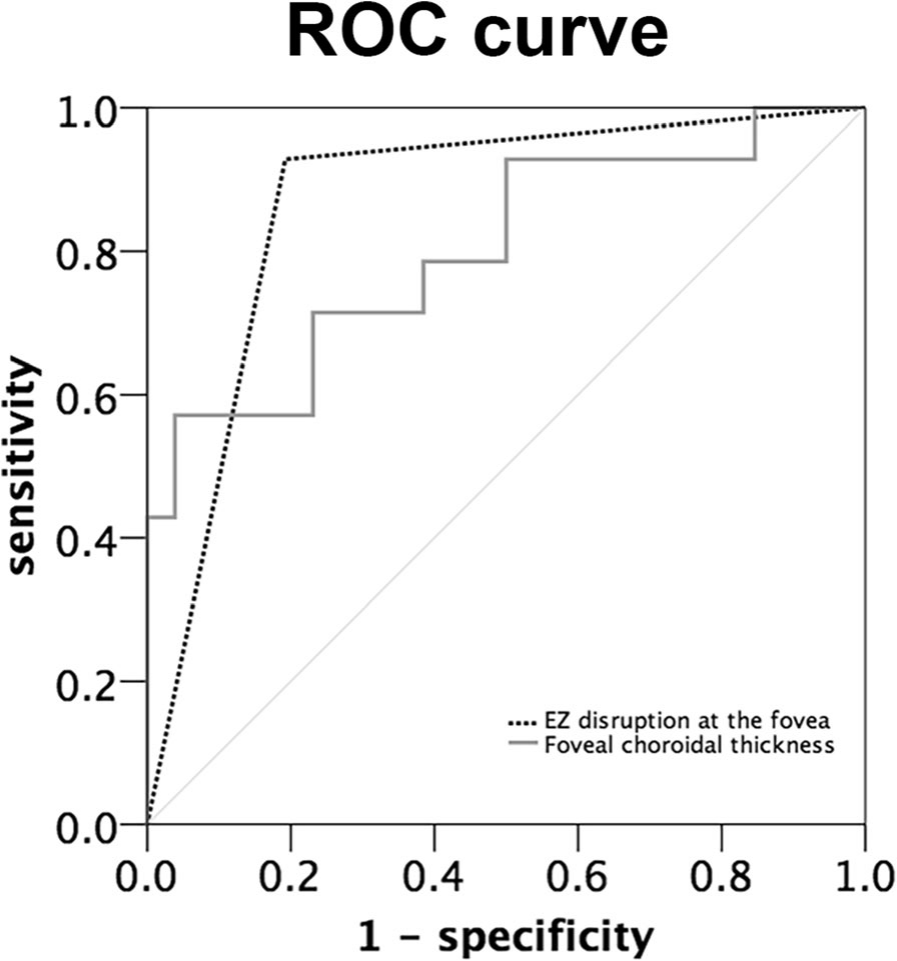
Receiver-operating characteristic (ROC) for two factors. “EZ disruption at fovea” (the dot line) showed the largest area under the curve (AUC) of 0.868, indicating the best discriminatory power. The AUCs of foveal choroidal thickness (the grey line) was 0.802 (*EZ* ellipsoid zone)

We performed a stepwise multivariate regression to determine the explanatory variables most strongly associated with legal blindness, including subfoveal choroidal thinning (*P* = 0.047) and EZ disruption at the fovea (*P* = 0.011) (Table 2). The image biometric that most sensitively predicted BCVA ≤20/200 in our study was EZ disruption at the fovea [Area under the curve (AUC): 0.868, 95% confidence interval (CI): 0.747–0.989]. The ROCs for EZ disruption at the fovea, and subfoveal choroidal thickness as predictors of BCVA ≤20/200 are shown in Fig. 6. We also analyzed the data using a continuous variable for BCVA (converted to logMAR for statistic analysis) instead of dividing the patients into two groups. The results were similar in that age, subfoveal choroidal thickness, EZ disruption at the fovea, crystalline deposits, and FAF stage 3 were significantly related to BCVA (supplementary file).

## Discussion

In this study, NIR was regarded as a convenient and noninvasive tool for the diagnosis of BCD because of its ability to detect crystalline deposits and sclerotic vessels in early and advanced stages of disease, respectively. We further explored the relationship between the features of multimodal retinal imaging with legal blindness (BCVA≤20/200). Notably, we found that EZ disruption at the fovea and decreased foveal choroidal thickness significantly correlated with legal blindness, while central retinal thickness, sclerotic vessels and FAF staging did not.

### NIR imaging

The most characteristic feature of BCD is the presence of retinal crystalline deposits first described by Dr. Bietti in 1937 [3]. As the disease progresses, the crystals diminish and disappear because of RPE atrophy and thinning of the Bruch’s membrane layer [14]. Pathologic analysis of crystalline deposits in BCD reported in 1987 revealed inclusion bodies in fibroblasts with lipid content within the crystalline deposits [15]. Oishi et al. reported that the presence of hyperreflective crystals on NIR has 100% sensitivity and 100% specificity for *CYP4V2* mutations among patients with chorioretinal dystrophy [7]. However, crystalline deposits were barely detectable in five legally blind eyes (group 1). Because the crystalline deposits decrease as the disease progresses, the absence of crystalline deposits on fundus photography could indicate more severe or advanced stages of BCD, which is usually accompanied by worse vision.

In addition to crystalline deposits, Dr. Bietti described “sclerotic vessels” in his three cases in 1937.^1^ The term sclerotic choroidal vessels refers to fibrotic, hyalinization, and occlusion of choroidal vessels. We found that large choroidal vessels in BCD are hyperreflective in NIR (Fig. 2). Kaiser-Kupfer et al. reported the thinned and diffuse fibrotic choroid with mixed with hypertrophic and hyperplastic melanocytes in pathology [16]. It is interesting to note that some retinal lesions such as lacquer cracks also presented as “hyperrefective” in NIR, which are associated with fibrotic tissue after the Bruch’s membrane ruptures in highly myopic eyes [17]. In our patients, all five legally blind eyes without obvious crystalline deposits had prominent sclerotic vessels on NIR. The large hyperreflective choroidal vessels on NIR are uncommon in other retinal degenerative conditions such as choroideremia or non-syndromic retinitis pigmentosa. Therefore, NIR imaging can be a useful tool to diagnose BCD as it is able to detect not only crystalline deposits in the early stages of disease but also sclerotic choroidal vessels in more advanced stages when crystalline deposits are invisible (Fig. 2).

### SD-OCT imaging

Several patients in our study had asymmetric BCVA between their two eyes even though image modalities showed similar crystalline deposits, similar FAF stage and pattern, prominent sclerotic vessels on NIR, and similar central retinal thickness. Notably, SD-OCT in these patients showed preserved central islands in the eye with better vision and destruction of the normal retinal lamella at the fovea in the legally blind eye (Fig. 4). The results of our statistical analysis revealed that EZ disruption at the fovea and thinner subfoveal choroid were strong indicators of legal blindness (Fig. 6). Central ellipsoid-preserved island could also be recognized on *En face* optical coherence tomography [18]. Miyata et al. used features of SD-OCT and OCTA to demonstrate a correlation between decreased BCVA and progression of VF defects [9]. Their results showed that a remaining central choroid capillary seen on OCTA, as well as foveal EZ disruption and choroid thickness seen on OCT, were well correlated with BCVA and VF. In this study, we analyzed the correlation of BCVA with the characteristics of other image modalities. Although fundus photo, NIR, and FAF can be used to monitor different stages of BCD [6, 8], we found that the characteristics on SD-OCT are still the strongest predictor for BCVA in BCD.

ORT was first described on SD-OCT in age related macular degeneration; however, it can be observed in other degenerative retinal disorders [19]. It has been suggested that ORT follows photoreceptor loss or retinal injuries in several retinal degeneration. However, it is not a unique feature of BCD. Because ORT structures were located outside the foveola on cross-sectional SD-OCT in BCD, their presence or absence is not related to BCVA in our study.

### FAF and SD-OCT correlation

Halfort et al. correlated FAF images with SD-OCT images in BCD patients, and found that a well-defined hypo-AF edge corresponded to abrupt outer retinal atrophy seen on SD-OCT [20]. However, in our study, we found that outer retinal atrophy or ellipsoid disruption on SD-OCT was not always correlated with hypo-AF (Fig. 4c). Although FAF staging has been shown to correlate well with fundus photo in disease progression, this staging has limitations for predicting legal blindness in BCD patients. There are several reasons why FAF imaging may not be an appropriate tool for predicting legal blindness. First, because of the lutein pigments, the FAF signals in the foveola are usually lower in the central fovea than in other areas. Therefore, FAF signals in the foveal area cannot be used as an indicator of RPE activity in areas outside the fovea. Second, the RPE is not the only cell type that contributes to AF in the retina: inflammatory cells such as macrophages and microglia recruited by debris in the outer segment may also contribute to AF or hyper-AF [21, 22]. Third, FAF detects mainly RPE activities. While hypo-FAF accurately indicates RPE atrophy, it is still challenging to determine whether areas with preserved AF signaling retain normal RPE activity (Fig. 4c). Although quantitative FAF has been used to study RPE activity in certain retinal diseases [23, 24], there are limitations to the application of quantitative FAF in BCD because of its irregular distribution and patterns of RPE atrophy.

### Limitations

There were several limitations to our study. First, the sample size was limited since BCD is not a common disease. Second, not all the patients in our study received genetic testing for *CYP4V2* mutations. Third, there is no standard for classifying the amount of crystals and sclerotic vessels detected; thus, quantification depends on image modalities and may be subjective. In turn, future software analysis of standard images to quantify these features may provide more information associated with visual function. Fourth, this is a cross-sectional clinical study based on different patients in different stages of disease. Long term follow-up of these patients may further help us to understand the nature of the primary defect as well as structural or functional changes associated with this disease. Fifth, OCTA analysis or *En face* OCT images were not performed in our patient. Instead, we investigated multiple image modalities including Color fundus, FAF, NIR and OCT to assess this disease.

## Conclusions

In conclusion, we found that NIR is a good diagnostic tool for BCD for detecting either crystalline deposits or sclerotic vessels. Furthermore, we suggest that central EZ disruption may be the best indicator for legal blindness as it can be assessed easily from SD-OCT images and correlates with BCVA. The resulting functional and structural correlation could help monitor disease progression in BCD and could be a good parameter for observing the effect of treatment in the future. Further study is required to identify ways of preventing the progression of this disease.

## Supplementary Information

**Additional file 1.**

## Data Availability

The dataset that used and analyzed during the current study are available from the corresponding author on reasonable request.

## Abbreviations

BCD: Bietti crystalline dystrophy
BCVA: Best corrected visual acuity
NIR: Near-infrared reflectance
FAF: Fundus autofluorescence
SD-OCT: Spectral domain-optical coherence tomography
EZ: Ellipsoid zone
*CYP4V2*: *Cytochrome P450 family 4 subfamily V member 2*
RPE: Retinal pigment epithelium
OCTA: Optical coherence tomography angiography
ORT: Outer retinal tubulation
ROC: Receiver-operating characteristic
AUC: Area under the curve
