# Supplementary material for: Predicting visual acuity in Bietti crystalline dystrophy: evaluation of image parameters
Source: BMC Ophthalmol. 2021 Feb 4;21:68. doi: 10.1186/s12886-021-01811-y (PMC7860191; doi:10.1186/s12886-021-01811-y)
Supplement: Supplementary file 1 — Additional file 1. [file 12886_2021_1811_MOESM1_ESM.docx]

| **Supplementary table. Simple linear regression of LogMAR and all clinical and imaging variables** | | | | | |  |  |
| --- | --- | --- | --- | --- | --- | --- | --- |
| **Variables** | Unstandardized Coefficients B | SD Error | Standardized Coefficients Beta | t | **p-value** | | |
| Age | 0.034 | 0.010 | 0.474 | 3.317 | **0.002** | | |
| Follow-up (months) | 0.005 | 0.005 | 0.174 | 1.090 | 0.283 | | |
| TVRM | 0.068 | 0.039 | 0.293 | 1.736 | 0.092 | | |
| Central retinal thickness (um) | -0.004 | 0.003 | -0.234 | -1.485 | 0.146 | | |
| Choroidal thickness (um) | -0.010 | 0.002 | -0.589 | -4.495 | **0.000** | | |
| Sex | 0.304 | 0.29 | 0.168 | 1.051 | 0.3 | | |
| Crystaline deposits | -1.025 | 0.397 | -0.387 | -2.584 | **0.014** | | |
| EZ disruption at fovea | 1.291 | 0.195 | 0.733 | 6.634 | **0.000** | | |
| Outer retinal tubulation | -0.478 | 0.274 | -0.273 | -1.747 | 0.089 | | |
| Sclerotic vessels | 0.564 | 0.420 | 0.213 | 1.343 | 0.187 | | |
| FAF staging |  |  |  |  |  | | |
| Stage 1 | -0.652 | 0.451 | -0.251 | -1.446 | 0.158 | | |
| Stage 2 | -0.285 | 0.330 | -0.153 | -0.862 | 0.395 | | |
| Stage 3 | 0.697 | 0.332 | 0.353 | 2.103 | **0.044** | | |
| SD: standard deviation; EZ: ellipsoid zone; FAF: fundus autofluorescence. *p<0.005 | | | | | | |  |
